# Genome Sequence of the Moderately Halophilic Yellow Sea Bacterium Lentibacillus salicampi ATCC BAA-719^T^

**DOI:** 10.1128/MRA.00702-19

**Published:** 2019-07-18

**Authors:** Milto Simoes Junior, Kyle S. MacLea

**Affiliations:** a Biotechnology Program, University of New Hampshire, Manchester, New Hampshire, USA; b Biology Program, University of New Hampshire, Manchester, New Hampshire, USA; c Department of Life Sciences, University of New Hampshire, Manchester, New Hampshire, USA; University of Delaware

## Abstract

Lentibacillus salicampi SF-20^T^ (=ATCC BAA-719^T^) was first isolated from a Yellow Sea salt field in Korea in 2002. Here, we report that the *L. salicampi* ATCC BAA-719^T^ genome sequence has a predicted length of 3,897,716 bp, containing 3,945 total genes and a CRISPR array, with a G+C content of 43.0%.

## ANNOUNCEMENT

Strains and species from the firmicute genus *Lentibacillus* have been identified in a number of salty environments (1–8). The type species of that genus, Lentibacillus salicampi ATCC BAA-719, was first collected in 2002 from a salt field of the Yellow Sea in Korea (1), and other strains of this species have also been identified in fermented fish sauces (2). *L. salicampi* SF-20^T^ (=ATCC BAA-719^T^) is described as a Gram-variable rod-shaped aerobic and motile bacterium capable of growing in 3% to 25% NaCl and forming spherical to oval endospores (1, 2). Characteristically, *L. salicampi* has meso-diaminopimelic acid as the diagnostic diamino acid in its peptidoglycan and has a cellular fatty acid profile that contains large amounts of branched fatty acids, particularly as anteiso-C_15:0_ and iso-C_16:0_ (1). Additionally, *L. salicampi* is closely related to species in other halophilic *Bacillaceae* genera, including *Virgibacillus*, *Gracilibacillus*, *Halobacillus*, *Filobacillus*, and *Pontibacillus* (1, 2, 9). As BAA-719 is the type strain of the *Lentibacillus* genus, its genome sequence is expected to be especially useful in resolving the assignment of new strains to the correct genera and for resolving taxonomic discrepancies among these halophilic bacilli. Here, we report the genome sequence of *L. salicampi* ATCC BAA-719^T^.

*L. salicampi* ATCC BAA-719^T^ was obtained from ATCC (Manassas, VA, USA) in a freeze-dried form, then rehydrated and cultured in marine broth 2216 (BD Difco, Billerica, MA, USA), and incubated at 30°C for 96 h at 1 atm. After rehydration, *L. salicampi* was grown at log phase before its genomic DNA (gDNA) was isolated using the QIAamp DNA mini kit (Qiagen, Valencia, CA, USA). Fragmentation of gDNA and attachment of sequence adapters were undertaken using the KAPA HyperPlus kit (KR1145, v.3.16; Wilmington, MA, USA) followed by sequencing on an Illumina HiSeq 2500 instrument (Hubbard Center for Genome Studies, Durham, NH, USA). Raw 250-bp reads (5,761,192 reads in total) were trimmed using Trimmomatic v.0.38 (settings were paired-end mode with a window size of 4, quality requirement of 15, and minimum read length of 36) and then assembled with the default parameters using SPAdes v.3.13.0 (10, 11). After the removal of small (<500-bp) contigs along with contaminants flagged with the NCBI Prokaryotic Genome Assembly Pipeline (PGAP) v.4.8 (below), QUAST v.5.0.2 (12) analysis verified 135 contigs—the largest being 483,654 bp—with an *N*_50_ value of 64,911 bp and a genome coverage of approximately 315×. PGAP (13, 14) provided gene identification and annotation. The assembled genome was 3,897,716 bp long, and PGAP revealed a total of 3,945 genes, 3,725 protein-coding sequences, 130 pseudogenes, 64 tRNAs, 21 copies of the rRNA genes, of which only the 5S rRNA gene is complete, 5 noncoding RNAs (ncRNAs), 1 CRISPR array, and a G+C content of 43.0%, in line with published values for the genus (42% to 44%) (3) and species (44% or 42.4%) (1, 2).

### Data availability.

The Lentibacillus salicampi ATCC BAA-719^T^ whole-genome shotgun sequence (WGS) project has been deposited at DDBJ/ENA/GenBank under the accession number SRHY00000000. The version described in this paper is the first version, SRHY01000000. The raw Illumina data from BioProject accession number PRJNA529678 were submitted to the NCBI Sequence Read Archive (SRA) under experiment accession number SRX5588714.
